# Supplementary material for: HIVprotI: an integrated web based platform for prediction and design of HIV proteins inhibitors
Source: J Cheminform. 2018 Mar 9;10:12. doi: 10.1186/s13321-018-0266-y (PMC5845081; doi:10.1186/s13321-018-0266-y)
Supplement: Supplementary file 2 — Additional file 2. Source code of HIVProtI web server. [file 13321_2018_266_MOESM2_ESM.zip › HIVprotI_Source-code/hivprotI/weka-3-6-6/doc/weka/core/Range.html]

Range


---


|  |  |  |
| --- | --- | --- |
| **Overview** \| **Package** \| **Class** \| **Tree** \| **Deprecated** \| **Index** \| **Help** \| **Weka's home** | |  |
| **PREV CLASS**   **NEXT CLASS** | **FRAMES**    **NO FRAMES**     **All Classes** |  |
| SUMMARY: NESTED \| FIELD \| CONSTR \| METHOD | DETAIL: FIELD \| CONSTR \| METHOD |  |


---


## weka.core Class Range

```
java.lang.Object
  weka.core.Range
```

**All Implemented Interfaces:**: java.io.Serializable, RevisionHandler

---

``` public class Range extends java.lang.Object implements java.io.Serializable, RevisionHandler ```

Class representing a range of cardinal numbers. The range is set by a
string representation such as:

`first-last
1,2,3,4` 

or combinations thereof. The range is internally converted from
1-based to 0-based (so methods that set or get numbers not in string
format should use 0-based numbers).

**Version:**
:   $Revision: 1.18 $

**Author:**
:   Len Trigg (trigg@cs.waikato.ac.nz)

**See Also:**: Serialized Form

---

**Constructor Summary**

`Range()`
  
          Default constructor.

`Range(java.lang.String rangeList)`
  
          Constructor to set initial range.


| **Method Summary** | |
| --- | --- |
| `boolean` | `getInvert()`             Gets whether the range sense is inverted, i.e. |
| `java.lang.String` | `getRanges()`             Gets the string representing the selected range of values |
| `java.lang.String` | `getRevision()`             Returns the revision string. |
| `int[]` | `getSelection()`             Gets an array containing all the selected values, in the order that they were selected (or ascending order if range inversion is on) |
| `static java.lang.String` | `indicesToRangeList(int[] indices)`             Creates a string representation of the indices in the supplied array. |
| `boolean` | `isInRange(int index)`             Gets whether the supplied cardinal number is included in the current range. |
| `static void` | `main(java.lang.String[] argv)`             Main method for testing this class. |
| `void` | `setInvert(boolean newSetting)`             Sets whether the range sense is inverted, i.e. |
| `void` | `setRanges(java.lang.String rangeList)`             Sets the ranges from a string representation. |
| `void` | `setUpper(int newUpper)`             Sets the value of "last". |
| `java.lang.String` | `toString()`             Constructs a representation of the current range. |

**Methods inherited from class java.lang.Object**

`equals, getClass, hashCode, notify, notifyAll, wait, wait, wait`

**Constructor Detail**

### Range

```
public Range()
```

:   Default constructor.

---


### Range

```
public Range(java.lang.String rangeList)
```

:   Constructor to set initial range.

    **Parameters:**: `rangeList` - the initial range **Throws:**: `java.lang.IllegalArgumentException` - if the range list is invalid


**Method Detail**

### setUpper

```
public void setUpper(int newUpper)
```

:   Sets the value of "last".

    :   **Parameters:**: `newUpper` - the value of "last"

---


### getInvert

```
public boolean getInvert()
```

:   Gets whether the range sense is inverted, i.e. all *except*
    the values included by the range string are selected.

    :   **Returns:**: whether the matching sense is inverted

---


### setInvert

```
public void setInvert(boolean newSetting)
```

:   Sets whether the range sense is inverted, i.e. all *except*
    the values included by the range string are selected.

    :   **Parameters:**: `newSetting` - true if the matching sense is inverted

---


### getRanges

```
public java.lang.String getRanges()
```

:   Gets the string representing the selected range of values

    :   **Returns:**: the range selection string

---


### setRanges

```
public void setRanges(java.lang.String rangeList)
```

:   Sets the ranges from a string representation. Note that setUpper()
    must be called afterwards for ranges to be actually set internally.

    :   **Parameters:**: `rangeList` - the comma separated list of ranges. The empty string sets the range to empty. **Throws:**: `java.lang.IllegalArgumentException` - if the rangeList was not well formed

---


### isInRange

```
public boolean isInRange(int index)
```

:   Gets whether the supplied cardinal number is included in the current
    range.

    :   **Parameters:**: `index` - the number of interest **Returns:**: true if index is in the current range **Throws:**: `java.lang.RuntimeException` - if the upper limit of the range hasn't been defined

---


### toString

```
public java.lang.String toString()
```

:   Constructs a representation of the current range. Being a string
    representation, the numbers are based from 1.

    :   **Overrides:**: `toString` in class `java.lang.Object`
    :   **Returns:**: the string representation of the current range

---


### getSelection

```
public int[] getSelection()
```

:   Gets an array containing all the selected values, in the order
    that they were selected (or ascending order if range inversion is on)

    :   **Returns:**: the array of selected values **Throws:**: `java.lang.RuntimeException` - if the upper limit of the range hasn't been defined

---


### indicesToRangeList

```
public static java.lang.String indicesToRangeList(int[] indices)
```

:   Creates a string representation of the indices in the supplied array.

    :   **Parameters:**: `indices` - an array containing indices to select. Since the array will typically come from a program, indices are assumed from 0, and thus will have 1 added in the String representation. **Returns:**: the string representation of the indices

---


### getRevision

```
public java.lang.String getRevision()
```

:   Returns the revision string.

    :   **Specified by:**: `getRevision` in interface `RevisionHandler`
    :   **Returns:**: the revision

---


### main

```
public static void main(java.lang.String[] argv)
```

:   Main method for testing this class.

    :   **Parameters:**: `argv` - one parameter: a test range specification


---


|  |  |  |
| --- | --- | --- |
| **Overview** \| **Package** \| **Class** \| **Tree** \| **Deprecated** \| **Index** \| **Help** \| **Weka's home** | |  |
| **PREV CLASS**   **NEXT CLASS** | **FRAMES**    **NO FRAMES**     **All Classes** |  |
| SUMMARY: NESTED \| FIELD \| CONSTR \| METHOD | DETAIL: FIELD \| CONSTR \| METHOD |  |


---
